# Pelvic organ prolapse: emerging concepts in molecular mechanisms and tissue repair

**DOI:** 10.3389/fcell.2026.1937362

**Published:** 2026-10-30

**Authors:** Zhenglin He, Hai Tong, Jiaxin Liu, Lubin Liu

**Affiliations:** 1 Department of Obstetrics and Gynecology, Chongqing Health Center for Women and Children, Chongqing, China; 2 Department of Obstetrics and Gynecology, Women and Children’s Hospital of Chongqing Medical University, Chongqing, China; 3 Department of Chemical and Biomolecular Engineering, University of California, Berkeley, CA, United States; 4 Department of Obstetrics and Gynecology, West China Hospital, Sichuan University, Chengdu, Sichuan, China

**Keywords:** adaptive repair, extracellular matrix, fibroblast state, mechanotransduction, pelvic floor reconstruction, pelvic organ prolapse, regenerative repair, tissue mechanics

## Abstract

Pelvic organ prolapse (POP) is a common and progressive pelvic floor disorder associated with pregnancy and vaginal delivery, ageing, obesity, menopause and other conditions that increase or prolong mechanical burden on pelvic supportive tissues. Previous studies have linked POP to extracellular matrix remodeling, oxidative stress, hormonal changes, genetic susceptibility and fibroblast dysfunction. However, these findings are still often presented as separate pathogenic theories or molecular observations, which makes it difficult to explain how mechanical challenge, cellular dysfunction and structural failure are biologically connected during disease progression. In this Review, we propose that POP can be more coherently understood along a mechanics–cell–matrix axis. Within this framework, chronic mechanical loading and impaired adaptive repair are regarded as the disease-setting in which prolapse develops. Mechanotransduction-related pathways are then considered the upstream bridge linking altered tissue mechanics to fibroblast state change. Oxidative stress, autophagy-related disturbance, apoptosis and senescence are further discussed as bridge-amplifier processes that deepen repair failure, whereas extracellular matrix imbalance is viewed as the structural endpoint of declining tissue support. Hormonal status, genetic susceptibility and immune remodeling further influence the course of POP. Fibrosis-associated fibroblast activation may coexist with collagen disorganization, matrix degradation and impaired elastic recoil. This review connects these tissue findings with the distinctive mechanical demands of pelvic support and considers their implications for repair (Tian et al., BJOG, 2024, 131, 1029–1041; Guler and Roovers, Biomolecules, 2022, 12, 94; Wu et al., Front. Med., 2024, 11, 1303044; Ruiz-Zapata et al., Sci. Rep., 2016, 6, 22971).

## 1 Introduction

Pelvic organ prolapse (POP) is a common and progressive pelvic floor disorder that can substantially impair daily activity, sexual function and quality of life (Deng et al., 2021). Pregnancy and vaginal delivery remain the best-recognized clinical risk factors, and ageing, menopause, obesity and other conditions associated with sustained increases in intra-abdominal pressure are also thought to contribute (Deng et al., 2021; Yu et al., 2022). Yet these factors alone do not fully explain why prolapse develops, why it often becomes clinically apparent years after the initiating insult, or why women with apparently similar exposures can follow very different disease courses (Deng et al., 2021; Wu et al., 2024).

Human and experimental studies have documented ECM remodeling, oxidative injury, hormonal effects and fibroblast dysfunction in POP, but their temporal relationships remain incompletely linked across anatomical scales (Deng et al., 2021; Habes et al., 2022; Guler and Roovers, 2022; Allen-Brady et al., 2022). The loading environment of the vaginal wall and apical supports provides a specific context for testing how these processes interact (Guler and Roovers, 2022; Wu et al., 2024).

Fibroblasts integrate mechanical cues with collagen turnover and elastic-fiber maintenance, while tissue strain and altered matrix architecture influence integrin- and cytoskeleton-associated signaling (Guler and Roovers, 2022; Wu et al., 2024; Ruiz-Zapata et al., 2016; Zhao et al., 2024). The resulting response may include excessive remodeling as well as impaired repair; it is not described adequately by collagen loss or fibrosis alone.

This Review examines the relationship between pelvic-specific loading, fibroblast behavior and ECM remodeling, with particular attention to the overlap between maladaptive repair and fibrosis. Table 1 organizes the evidence as Pathogenic setting, Upstream bridge, Central effector layer, Bridge-amplifier layer, Structural endpoint and Modifier layers. The translational implications are considered in the context of these distinct evidence sources.

**TABLE 1:** Mechanistic layers in the mechanics-cell-matrix framework for pelvic organ prolapse (Deng et al., 2021; Tian et al., 2024; Guler and Roovers, 2022; Wu et al., 2024).

Layer	Representative readout	Typical evidence base	Interpretive role in this review	References
Pathogenic setting	Pregnancy, delivery, ageing, menopause, elevated intra-abdominal pressure	Clinical and epidemiological evidence	Defines chronic loading and impaired adaptive repair	Deng et al. (2021); Yu et al. (2022); Wang et al. (2018); Zeng et al. (2020)
Upstream bridge	Matrix stiffness, integrin-related sensing, cytoskeletal tension, TGF-beta and p38-linked signaling	Cell and tissue mechanistic studies	Connects tissue stress to cell-state change	Wu et al. (2024); Zhao et al. (2024); Kong et al. (2025); Wang X. et al. (2025); Quiles et al. (2024)
Central effector layer	Fibroblast activation, myofibroblast-like transition, impaired repair capacity	Human tissue plus fibroblast functional studies	Explains how cellular dysfunction emerges	Guler and Roovers, (2022); Fan et al. (2023); Zhu et al. (2024); Zhou Z. et al. (2025); Li et al. (2023); Wang et al. (2022)
Bridge-amplifier layer	Oxidative stress, autophagy disturbance, apoptosis, senescence	Cellular and stress-pathway studies	Explains persistence and amplification of repair failure	Habes et al. (2022); Wang et al. (2024); Yu et al. (2023); Yan et al. (2025); Hare et al. (2021)
Structural endpoint	Collagen imbalance, elastin disruption, MMP/TIMP dysregulation	Dense human tissue and mixed-model evidence	Represents tissue-support failure	Tian et al. (2024); Saputra et al. (2022); Saputra et al. (2024); Xiong et al. (2025); Rui et al. (2025); Wang Q. et al. (2025); L et al. (2019)
Modifier layers	Hormones, genetics, omics, immune remodeling	Association, omics and selected functional studies	Explain heterogeneity and future stratification	Allen-Brady et al. (2022); Jiang et al. (2025); Chen et al. (2024); Yu et al. (2022); Tchoukalova et al. (2025); Li et al. (2021b); Zhao et al. (2023); Wei et al. (2026)

## 2 Methods

Relevant publications were identified through PubMed, Google Scholar and the reference lists of clinical, histopathological and mechanistic POP studies. Search terms included pelvic organ prolapse, vaginal wall, uterosacral ligament, fibroblast, extracellular matrix, fibrosis, mechanotransduction, biomechanics, oxidative stress, autophagy, apoptosis, senescence, inflammation, genetics and regenerative repair. This is a narrative review, not a systematic review or meta-analysis.

The synthesis distinguishes clinical/histopathological observations, *ex vivo* mechanical measurements, primary human fibroblast and controlled-loading experiments, animal models, and transcriptomic or single-cell analyses. Each source was considered according to the tissue or model examined, the intervention performed and the inference supported by the data (Guler and Roovers, 2022; Wu et al., 2024; Ruiz-Zapata et al., 2016; Fan et al., 2023; Jennings et al., 2024; Li et al., 2021a; Li et al., 2021b; Hare et al., 2021; Dubik et al., 2025; Zhou Q. et al., 2025).

## 3 From risk factors to a pathogenic setting: chronic mechanical loading and impaired adaptive repair

Vaginal delivery may damage levator ani muscles, fascial attachments and the vaginal wall, creating defects in a support system that subsequently experiences recurrent loading (Deng et al., 2021; Wu et al., 2024; Wang et al., 2018). Changes in intra-abdominal pressure during coughing, lifting and defecation translate into tensile and shear forces across the vaginal wall and apical supports. The extracellular matrix must sustain these cyclic, multidirectional loads while retaining compliance and recoil. Collagen fiber organization contributes tensile resistance; FBLN5- and LOXL1-associated elastic-fiber homeostasis contributes recovery after deformation (Saputra et al., 2022; Saputra et al., 2024; Zhang R. et al., 2024; Li et al., 2021a).

The pelvic floor is a load-bearing system that must continually adapt to fluctuations in abdominal pressure and to the mechanical demands of daily life (Wu et al., 2024). Under physiological conditions, this does not necessarily result in failure, because connective tissue remodeling and cellular repair can preserve tissue integrity (Wu et al., 2024). The problem arises when loading becomes excessive, repetitive or prolonged and the reparative response is no longer sufficient to restore mechanical competence (Wu et al., 2024; Ruiz-Zapata et al., 2016). In that setting, the critical issue is not exposure to force alone, but whether the tissue retains the capacity for adaptive repair (Guler and Roovers, 2022; Wu et al., 2024).

Repeated loading becomes injurious when microdamage accumulates faster than the tissue can restore collagen architecture and elastic-fiber integrity. Structural changes then redistribute strain within the vaginal wall and uterosacral support, generating local mechanical cues to which resident cells respond (Wu et al., 2024; Ruiz-Zapata et al., 2016; Zhao et al., 2024; Li et al., 2021a). Figure 1 illustrates these linked mechanical and cellular processes.

**FIGURE 1:**
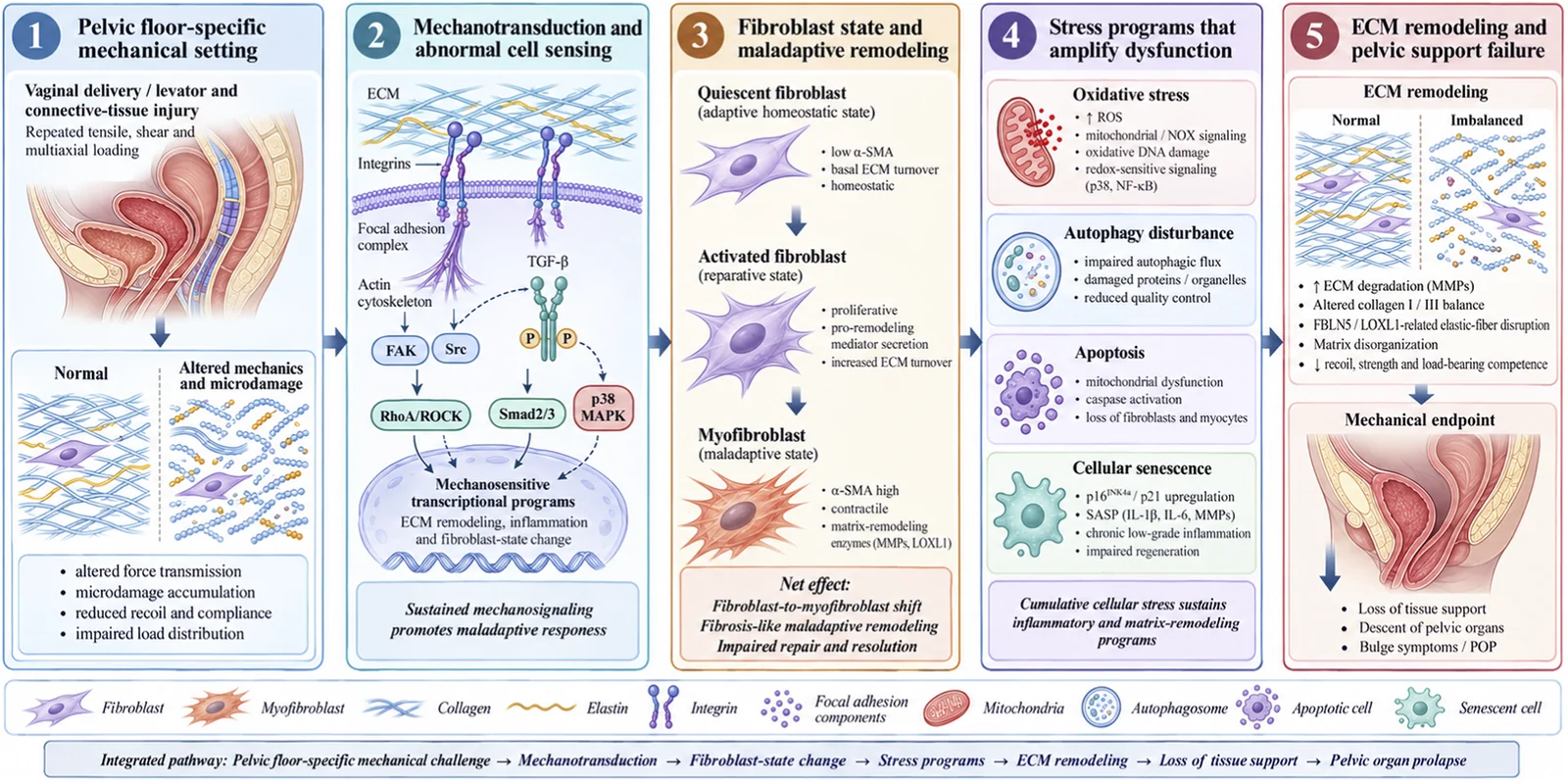
Pelvic floor-specific mechanics–cell–matrix framework in POP. Obstetric and connective-tissue injury, repetitive tensile and shear loading, abnormal mechanotransduction, fibroblast-state changes and cellular stress converge on ECM remodeling and pelvic support failure. This conceptual schematic synthesizes published findings; some visual elements were developed with AI-assisted illustration, and the final graphic was recreated and refined in Adobe Illustrator (Adobe Inc.). No original experimental data are shown (Deng et al., 2021; Guler and Roovers, 2022; Wu et al., 2024; Ruiz-Zapata et al., 2016; Zhao et al., 2024; Kong et al., 2025; Wang et al., 2024; Saputra et al., 2022; Saputra et al., 2024; Zhang R. et al., 2024; Li et al., 2021a; Wang et al., 2018; Zeng et al., 2020).

## 4 Mechanotransduction as the upstream bridge from tissue stress to cell-state change

Mechanically controlled experiments have begun to identify how loading regulates pelvic-support fibroblasts. Wang et al. exposed primary vaginal fibroblasts from postmenopausal women with and without POP to uniaxial cyclic stretch (0.1 Hz, 10% elongation) and assessed extracellular polymer architecture together with COL I, COL III and small leucine-rich proteoglycans. Stretching altered the organization of cell-associated matrix, with disrupted networks observed in POP-derived cultures (Wang et al., 2018). Experiments combining mechanical stress and hydrogen peroxide in uterosacral-ligament fibroblasts further linked physical and oxidative inputs to TGF-β1-associated MMP/TIMP dysregulation (Zhang et al., 2017).

Matrix rigidity provides a second, distinct experimental input. Substrate-composition and stiffness studies using vaginal fibroblasts have linked the mechanical environment to myofibroblast differentiation and ECM production (Wang X. et al., 2025; Ruiz-Zapata et al., 2020). Zhao et al. identified a more specific route in stiffness-controlled L929 fibroblasts: autophagy-dependent redistribution of Kindlin-2 promoted MOB1 degradation and nuclear YAP signaling, while autophagy inhibition partially reversed the myofibroblast response (Zhao et al., 2024). Increased cytoplasmic Kindlin-2 and nuclear YAP were also observed in POP tissues, but the perturbation experiments were performed in a fibroblast cell line rather than in intact human pelvic-support tissue (Zhao et al., 2024). Studies of vaginal fibroblast differentiation on stiffness-controlled matrices (Ruiz-Zapata et al., 2020) and METTL3-associated stiffness responses (Wang X. et al., 2025) provide a complementary, tissue-relevant cell context, although neither reconstructs the multidirectional loading of the whole pelvic floor.

Integrin–focal-adhesion signaling, RhoA/ROCK-mediated cytoskeletal tension and TGF-β/SMAD or p38 responses provide candidate routes from loading to matrix remodeling (Wu et al., 2024; Kong et al., 2025; Wang X. et al., 2025). Their interpretation depends on the model: a stretch device tests imposed strain, a stiffness-controlled substrate tests resistance to cell-generated force, and patient histology documents the established tissue phenotype. These systems answer different questions and should not be treated as interchangeable evidence of a single temporal sequence. Figure 2 summarizes the histopathological, biomechanical and experimental evidence discussed above.

**FIGURE 2:**
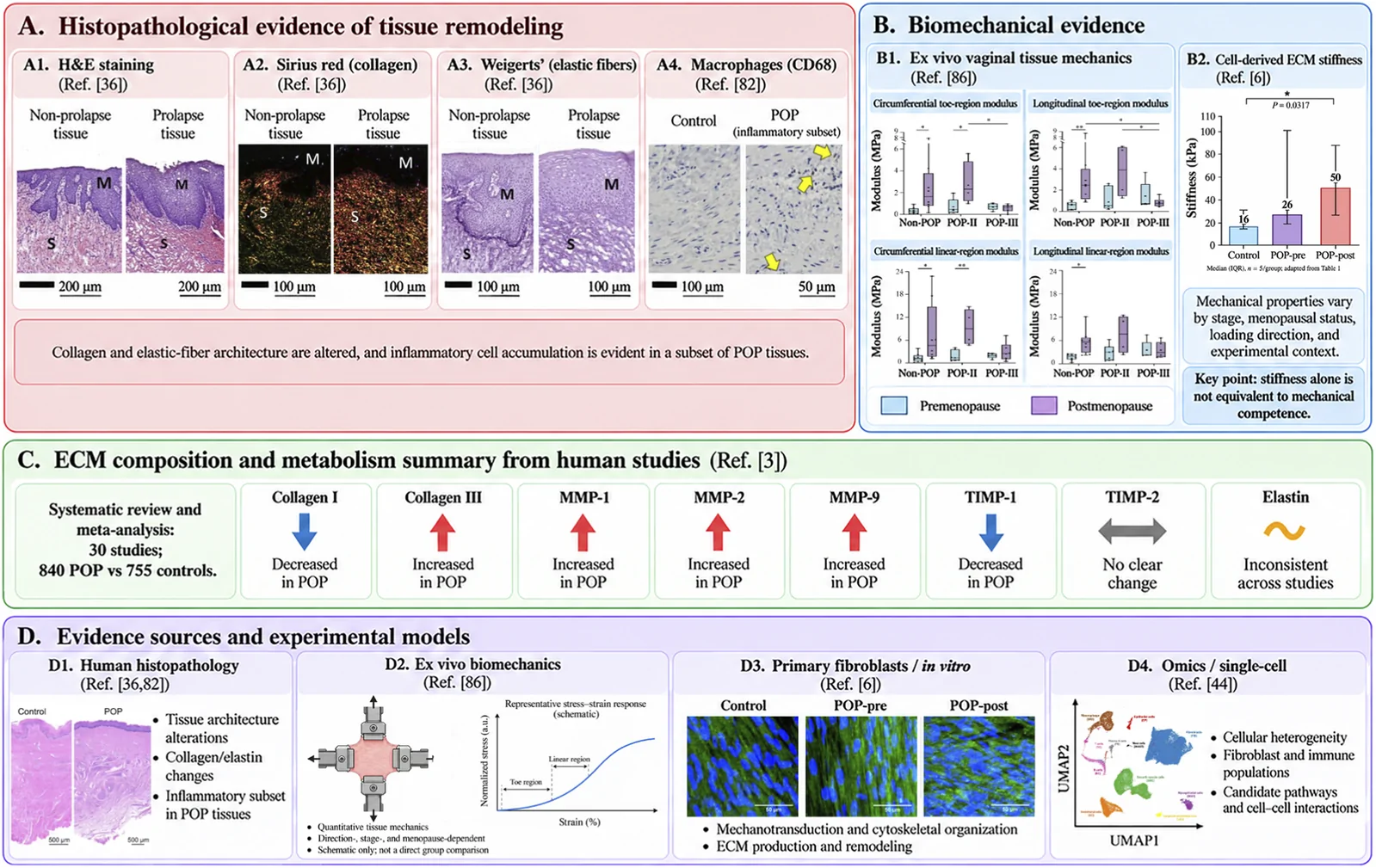
Published histopathological, biomechanical and experimental evidence of heterogeneous remodeling in POP. **(A)** H&E, Sirius red and Weigert-staining images were selected and rearranged from Li et al. (2021a); macrophage-related histology was selected from Orlicky et al. (2024). **(B)** Human biaxial biomechanical findings are adapted from Zhou Q. et al. (2025); cell-derived ECM Young’s modulus values are taken from Ruiz-Zapata et al. (2016) (medians with reported interquartile ranges, n = 5 per group). **(C)** Collagen, MMP/TIMP and elastin trends summarize the meta-analysis of Tian et al. (2024). **(D)** Published human histopathology (Li et al., 2021a; Li et al., 2021b), fibroblast-generated matrix images (Ruiz-Zapata et al., 2016) and single-cell profiles (Li et al., 2021b) are assembled alongside an illustrative biaxial-test schematic (not experimental group-comparison data). All image panels and measurements derive from published sources; the present figure is a secondary synthesis, not newly acquired experimental data.

## 5 Fibroblast state as the central effector layer in pelvic organ prolapse

Fibroblasts are among the most abundant and functionally important stromal cells in pelvic supportive connective tissues (Guler and Roovers, 2022; Fan et al., 2023). They are responsible for maintaining collagen turnover, elastin integrity and broader matrix homeostasis, and are therefore well positioned to shape how tissues respond to injury over time (Guler and Roovers, 2022; Zhao et al., 2024; Fan et al., 2023). In POP, fibroblast-related abnormalities have been reported consistently across human tissues, cell models and experimental systems (Guler and Roovers, 2022; Fan et al., 2023; Zhu et al., 2024; Guo et al., 2023). This makes them more than just 1 cell type among many: they represent a cellular level at which multiple pathogenic signals begin to converge (Guler and Roovers, 2022; Wu et al., 2024; Wang et al., 2024).

Physiological fibroblast activation supports matrix renewal after injury, whereas persistent myofibroblast differentiation can maintain contractility and abnormal matrix turnover. POP-related fibroblast studies document altered collagen regulation, apoptosis and stiffness responses alongside increased matrix degradation in human support tissues (Guler and Roovers, 2022; Zhao et al., 2024; Wang et al., 2024; Fan et al., 2023; Jin et al., 2024; Yin et al., 2020). Abnormal repair therefore concerns matrix composition, organization and durability, not simply the amount of collagen produced.

Fibroblast activation in POP shares features with fibrosis, including myofibroblast differentiation, TGF-β/p38-related signaling, increased contractility and stiffness-sensitive matrix production (Guler and Roovers, 2022; Zhao et al., 2024; Wang X. et al., 2025; Ruiz-Zapata et al., 2020). In classical organ fibrosis, these programs commonly produce persistent scar-like ECM accumulation. POP has a less uniform structural phenotype: pooled human studies show reduced type I collagen, increased type III collagen and elevated MMP-1/-2/-9 activity, alongside elastic-fiber abnormalities (Tian et al., 2024; Saputra et al., 2022; Saputra et al., 2024; Li et al., 2021a). Fibrosis-like remodeling is therefore part of POP pathobiology, but excessive collagen deposition does not adequately define the clinical disease.

## 6 Oxidative stress, autophagy, apoptosis and senescence as stress-intensifying processes

Oxidative stress has both systemic and local components in POP. Ageing and endocrine or metabolic changes can reduce antioxidant reserve, whereas mechanically strained pelvic fibroblasts can generate local redox stress (Habes et al., 2022; Zhang et al., 2017; Liu et al., 2016; Kim et al., 2013). In cultured uterosacral-ligament fibroblasts, hydrogen peroxide altered collagen metabolism and the MMP/TIMP balance; combined mechanical and oxidative exposure implicated TGF-β1-associated signaling (Zhang et al., 2017; Liu et al., 2016). More directly, ATF3 silencing reduced H2O2-associated ROS, apoptosis, MMP2/MMP9 expression and ECM loss through p38/NRF2-related signaling in vaginal fibroblasts (Feng et al., 2021). SGK1-dependent regulation of GSK-3β/Fyn/NRF2 also limited oxidative injury and matrix degradation in human uterosacral-ligament fibroblast models (Yan et al., 2025). These findings support a reciprocal relationship between local cellular stress and matrix remodeling, rather than assigning oxidative injury exclusively to ageing or to mechanical trauma.

Autophagy contributes to this response through the regulation of cellular quality control and fibroblast phenotype. In vaginal fibroblasts, elevated miR-19-3p was associated with reduced IGF-1/COL I expression and increased autophagy and apoptosis through inhibition of AKT/mTOR/p70S6K signaling (Yin et al., 2021). Separately, stiffness-induced autophagy promoted Kindlin-2 redistribution and myofibroblast conversion in L929 cells (Zhao et al., 2024). The first study implicates reduced AKT/mTOR-associated signaling in enhanced autophagy and diminished collagen production, whereas the second links a stiffness-induced autophagic response to a contractile myofibroblast state. Autophagy thus has context-dependent effects on matrix-producing cells; neither finding justifies a general assumption that increasing or suppressing autophagy would benefit all POP tissues.

Apoptotic pathways affect the pool of cells available for repair. Human POP tissue and fibroblast studies have implicated mitochondrial oxidative injury, while experimentally induced hypoxia and advanced glycation end-product exposure activated apoptosis-related pathways in uterosacral-ligament fibroblasts (Kim et al., 2013; Sima et al., 2024; Zhao et al., 2018; Sima et al., 2022). AGE–RAGE/MAPK/NF-κB signaling has also been linked to reduced collagen I expression in vaginal fibroblasts (Chen et al., 2017). Excess apoptosis may deplete cells needed for maintenance of collagen and elastic-fiber networks, whereas surviving cells can still adopt a matrix-remodeling phenotype. Comparing viability assays with collagen production, matrix degradation and contractile markers is therefore more informative than using apoptosis as a stand-alone proxy for tissue weakening.

Senescence may sustain remodeling even when fibroblasts survive. POP-derived human uterosacral-ligament fibroblasts exhibited greater senescence and ROS burden together with lower SIRT1 expression; pharmacological SIRT1 activation attenuated these phenotypes in culture (Wang et al., 2024). Senescence-associated secretory proteins have also been measured in vaginal secretions, while vaginal-distension and Fbln5-deficient mouse models examine injury-associated changes in senescence markers (Sawyer et al., 2023; Hare et al., 2021). In the human fibroblast study, the senescent-cell fraction was 43.63% ± 10.62% in POP-derived cultures versus 4.84% ± 5.32% in control cultures; SIRT1 activation reduced the senescence and ROS signals (Wang et al., 2024). These observations link cellular ageing, local injury and repair capacity, although secretory markers and acute injury models do not by themselves establish the temporal course of human POP.

## 7 Extracellular matrix imbalance as a structural endpoint and feedback mechanism in tissue-support failure

The extracellular matrix (ECM) is a major determinant of pelvic floor strength, resilience and load-bearing capacity (Tian et al., 2024; Saputra et al., 2022; Saputra et al., 2024; Sridharan et al., 2012). Its principal components, particularly collagen and elastic fibers, are essential for maintaining tissue integrity and mechanical support (Tian et al., 2024; Saputra et al., 2022; Saputra et al., 2024). For this reason, ECM imbalance has long been recognized as a major pathological feature of POP (Tian et al., 2024). Previous studies have repeatedly linked prolapse to disturbed collagen homeostasis, abnormal elastic-fiber maintenance and increased matrix degradation (Tian et al., 2024; Zhu et al., 2021; Saputra et al., 2022; Saputra et al., 2024; Sun et al., 2016). Read on their own, these findings might appear to describe only the structural consequences of disease. Taken together, however, they also suggest the point at which earlier mechanical and cellular disturbances become materially embedded in tissue architecture (Wu et al., 2024; Ruiz-Zapata et al., 2016).

Local matrix stiffness must be separated from organ-level support function. In fibroblast-generated ECM, median effective Young’s modulus was 16 kPa for controls, 26 kPa for premenopausal POP and 50 kPa for postmenopausal POP (n = 5 per group); the control–POP-post difference was significant (P = 0.0317) (Ruiz-Zapata et al., 2016). These values describe cell-derived matrices, not intact vaginal tissue. Planar biaxial testing of human vaginal tissue has demonstrated menopause-, stage- and direction-dependent moduli, including reduced toe-region moduli in postmenopausal stage-III prolapse relative to selected comparator groups (Zhou Q. et al., 2025). Mechanical competence also depends on recoil, extensibility and resistance to repetitive loading.

At the molecular level, the evidence remains centered on a familiar set of ECM-related changes. POP has repeatedly been associated with imbalance between matrix synthesis and degradation, with matrix metalloproteinases and their inhibitors representing one of the best-studied regulatory axes (Tian et al., 2024; Chen et al., 2014; Xue et al., 2025; Leegant et al., 2015; Tola et al., 2018; Vulic et al., 2011). Molecules involved in elastic-fiber biology, especially FBLN5 and LOXL1, have also received sustained attention because of their roles in preserving tissue elasticity and recoil (Zhang R. et al., 2024; Li et al., 2021a; Babinski et al., 2022; Ma et al., 2012). Once these systems become disrupted, the pelvic floor may gradually lose not only tensile strength but also the mechanical responsiveness needed to recover from repeated strain (Tian et al., 2024; Li et al., 2021a).

Proteomics and label-free structural imaging now complement collagen-expression and MMP/TIMP studies with information about tissue architecture (Jennings et al., 2024; Thomas et al., 2025; Wang Q. et al., 2025; Sun et al., 2015; Sridharan et al., 2012; L et al., 2019). Together with mechanical testing, these methods help distinguish net matrix composition from how matrix fibers are organized and transmit load. They also provide outcome measures for hydrogel, scaffold and exosome-based regenerative approaches (Zhou et al., 2024; Miller et al., 2023; Zhang D. et al., 2024; Mao et al., 2021).

## 8 Modifiers of the pathogenic cascade: hormones, genetic susceptibility, and the immune microenvironment

Hormones, inherited connective-tissue variation and immune-cell activity modify the matrix response to injury. Their effects may differ among pelvic compartments and with age, making them important contributors to POP heterogeneity (Allen-Brady et al., 2022; Jiang et al., 2025; Chen et al., 2024; Yu et al., 2022; Tchoukalova et al., 2025; Li et al., 2021b; Zhao et al., 2023; Wei et al., 2026).

Hormonal context provides one of the clearest examples. Estrogen deficiency has long been linked to pelvic floor vulnerability, particularly after menopause, and both experimental and clinical studies suggest that endocrine background can affect fibroblast behavior, collagen metabolism and tissue repair capacity (Yu et al., 2022; Xie et al., 2022; Tyagi et al., 2019). Genetic susceptibility adds a related but distinct layer. Variants linked to connective-tissue integrity, elastic-fiber assembly, hormone metabolism and oxidative-stress regulation suggest that different individuals may enter the same mechanical environment with different biological reserve (Allen-Brady et al., 2022; Jiang et al., 2025; Li et al., 2022). Epigenetic regulation may further shape this response, although the current evidence remains more associative than functionally resolved (Jiang et al., 2025; Wang X. et al., 2025; Zhao et al., 2023).

Single-cell and transcriptomic studies identify heterogeneous fibroblast, smooth-muscle and immune-cell populations in POP tissues and generate candidates for functional testing (Fan et al., 2023; Yu et al., 2023; Rui et al., 2025; Li et al., 2021b). Human histopathology also demonstrates that inflammatory infiltrates are not universal: Orlicky and colleagues observed macrophage and other immune-cell enrichment in an inflammatory subset of prolapsed uterosacral ligaments (Orlicky et al., 2024). Adler et al. described macrophage-rich “hot” and macrophage-poor “cold” fibrosis in a tissue-repair cell-circuit model (Adler et al., 2020). These concepts offer a comparison for POP inflammatory heterogeneity, without equating distinct cross-sectional tissue phenotypes with confirmed stages of a single process.

## 9 Translational implications: repair strategies informed by the pathogenic chain

POP treatment relies principally on symptom management and anatomical support. Regenerative approaches address a different question: whether a repair construct can restore load-bearing performance while supporting collagen and elastic-fiber organization and appropriate fibroblast activity (Deng et al., 2021; Wu et al., 2024; Miller et al., 2023).

Mechanically informed or load-aware materials should be assessed for compliance matching, anisotropy, viscoelasticity, load transfer and resistance to repeated deformation, not tensile strength alone. An injectable fibrous hyaluronic-acid hydrogel used to augment uterosacral ligament suspension in rats improved the 24-week load at failure from 0.89 ± 0.28 N for suspension alone to 1.37 ± 0.31 N with hydrogel augmentation; the intact ligament reached 1.70 ± 0.36 N (Miller et al., 2023). This illustrates how a pelvic-support-specific animal repair model can provide a functional mechanical endpoint, although it does not establish long-term clinical efficacy. In preclinical evaluation, load at failure should be considered alongside tissue compliance and repeated-load behavior, because a construct can resist one tensile test yet transfer nonphysiological strain to neighboring supports.

Biological repair studies provide complementary endpoints. TGF-beta1-induced smooth muscle differentiation of muscle-derived stem cells has been examined (Tang et al., 2019), and basic fibroblast growth factor overexpression has been investigated in stem cells for pelvic tissue regeneration (Wu et al., 2022). Recombinant humanized collagen III increased proliferation, migration and ECM synthesis while reducing senescence in POP-derived vaginal fibroblasts; in a rat simulated-birth-injury model, treatment was associated with higher collagen and angiogenesis-related markers (Zhang et al., 2025). TNF-α-conditioned mesenchymal-stem-cell exosomes improved selected vaginal-wall ECM and inflammatory markers in rats with pelvic floor dysfunction (Zhou et al., 2024). Puerarin-loaded electrospun patches combined sustained release with anti-inflammatory and pro-collagen effects, but their *in vivo* testing used a rat abdominal-wall defect rather than a POP repair model (Zhang D. et al., 2024). These different models establish biological plausibility, not equivalent evidence of durable pelvic support.

Stress-directed strategies are at an earlier stage. Antioxidant and NRF2-related interventions have modified redox injury and MMP/TIMP regulation in pelvic fibroblast experiments (Feng et al., 2021; Yan et al., 2025), and SIRT1 activation has reduced oxidative stress and senescence in cultured uterosacral-ligament fibroblasts (Wang et al., 2024). Autophagy-targeting interventions remain dependent on cell state and mechanical context, whereas senolytic effects have mainly been explored in experimental vaginal fibroblast systems (Zhao et al., 2024; Yin et al., 2021; Florian-Rodriguez et al., 2022). Such agents should be evaluated against tissue-quality and mechanical outcomes before being combined with reconstructive materials. Table 2 summarizes these intervention directions without treating their evidence levels as equivalent.

**TABLE 2:** Potential translational entry points suggested by the mechanics-cell-matrix framework (Guler and Roovers, 2022; Wu et al., 2024; Chen et al., 2024).

Mechanistic node	Candidate intervention direction	Expected biological effect	Current maturity	References
Mechanical environment	Load-aware biomaterials, mechanically compatible support constructs	Reduce abnormal force transmission and preserve tissue compliance	Conceptual to preclinical	Wu et al. (2024); Wang X. et al. (2025); Quiles et al. (2024); Mao et al. (2021)
Fibroblast state	Fibroblast-state normalization strategies	Improve adaptive repair and normalize matrix turnover	Preclinical	Guler and Roovers, (2022); Fan et al. (2023); Zhang et al. (2025); Li et al. (2023); Wang et al. (2022); Rathnayake et al. (2023)
Stress programs	Antioxidant, pro-survival or autophagy-modulating strategies	Limit cellular exhaustion and preserve reparative capacity	Preclinical to exploratory	Habes et al. (2022); Wang et al. (2024); Yan et al. (2025); Li et al. (2018)
ECM quality	Matrix-supportive regenerative materials, hydrogel or scaffold-based repair	Improve collagen/elastin organization and structural performance	Preclinical	Tian et al. (2024); Saputra et al. (2022); Saputra et al. (2024); Zhou et al. (2024); Miller et al. (2023); Zhang D. et al. (2024); Mao et al. (2021)
Integrated strategy	Combined force-cell-matrix repair design	Reduce recurrence and improve durable tissue support	Future translational priority	Guler and Roovers, (2022); Wu et al. (2024); Chen et al. (2024); You et al. (2025); Tchoukalova et al. (2025); Zhao et al. (2021); Miller et al. (2023)

## 10 Discussion

POP combines features often separated into degenerative and fibrotic categories. Studies of human support tissues report collagen I/III imbalance, elevated MMP activity and elastic-fiber disruption, while fibroblast experiments demonstrate TGF-β-related activation and stiffness-sensitive myofibroblast differentiation (Tian et al., 2024; Guler and Roovers, 2022; Zhao et al., 2024; Wang X. et al., 2025; Ruiz-Zapata et al., 2020). Fibrosis-related signaling and structural weakening can therefore coexist. The clinically defining outcome is loss of pelvic support, not necessarily net accumulation of scar-like matrix.

Biomechanical findings further distinguish matrix state from load-bearing performance. *In vitro* ECM stiffness, *ex vivo* tissue moduli and *in vivo* compliance describe different levels of organization and loading conditions (Ruiz-Zapata et al., 2016; Dubik et al., 2025; Feola et al., 2013; Epstein et al., 2007; Garcia-Mejido et al., 2024; Zhou Q. et al., 2025). The divergent results are informative: local matrix stiffening can accompany reduced tissue recoil or altered directional behavior. Reporting only “increased stiffness” obscures these differences and overlooks the fatigue and multiaxial loading demands of pelvic support.

The inflammatory component is also heterogeneous. The macrophage-rich histopathological subset described in uterosacral ligament specimens suggests that immune–stromal interactions could influence tissue repair in selected patients (Orlicky et al., 2024). However, cross-sectional surgical samples cannot resolve when these interactions arise or whether they persist. Mechanistic cell-circuit models of hot and cold fibrosis provide testable questions about macrophage–fibroblast interactions rather than a diagnostic classification for POP (Adler et al., 2020).

Priority studies should pair anatomically matched tissue histology with tensile and shear testing, incorporate menopausal status and POP stage, and compare molecular phenotypes using spatial or single-cell methods. Longitudinal sampling around pelvic injury or early prolapse, together with multiaxial organotypic models, would help establish how oxidative stress, fibroblast activation, senescence and matrix failure interact over time (Fan et al., 2023; Li et al., 2021b; Thomas et al., 2025; Hare et al., 2021; Dubik et al., 2025; Zhou Q. et al., 2025).

## 11 Conclusion

The available evidence identifies POP as a disorder of mechanically ineffective matrix remodeling in a pelvic support system exposed to repeated deformation. Fibrosis-associated fibroblast activity can occur alongside collagen imbalance, elastic-fiber failure and increased matrix degradation. These findings explain why local stiffness and overall tissue-support capacity need not move in the same direction. Mechanically informed reconstruction should therefore aim to restore organized load transfer, elastic recovery and reparative cell function, while stress-targeting approaches require evaluation in biologically and mechanically relevant POP models.
